# Interspinous process stabilization with Rocker via unilateral approach versus X-Stop via bilateral approach for lumbar spinal stenosis: a comparative study

**DOI:** 10.1186/s12891-015-0786-9

**Published:** 2015-11-01

**Authors:** Weimin Huang, Zhengqi Chang, Jingtao Zhang, Ruoxian Song, Xiuchun Yu

**Affiliations:** Department of Orthopaedics, General Hospital of Jinan Military Commanding Region, NO.25 Shifan Road, Jinan, Shandong 250031 People’s Republic of China

**Keywords:** Rocker, X-Stop, Interspinous process stabilization, Lumbar spinal stenosis

## Abstract

**Background:**

Rocker is a novel interspinous process stabilization (IPS) that can be installed via unilateral approach by virtue of its unique design. This controlled study compared the clinical outcome of Rocker versus X-Stop to access the feasibility and validity of the novel IPS.

**Methods:**

From March 2011 to September 2012, 32 patients treated with Rocker and 30 patients treated with X-Stop were enrolled in this study. The primary clinical outcome measure was Oswestry Disability Index (ODI) score. The secondary clinical outcome measure was Japanese orthopaedics association (JOA) score. Disc height index (DHI) and foraminal height index (FHI) were measured for postoperative radiographic evaluation. Implant failures were also recorded.

**Results:**

There were 55 patients with complete data during 24 months follow-up. Among the 55 patients, 38 patients underwent IPS in combination with microdecompression. At the final follow-up, 49 patients achieved a minimal clinical important difference (≥8 points ODI improvement). The mean operative time was 53.6 min (range, 30 to 90 min) in Rocker group and 63.1 min (range, 30 to 100 min) in X-Stop group. The average blood loss was 111 ml (range, 50 to 400 ml) in Rocker group and 138 ml (range, 50 to 350 ml) in X-Stop group. ODI score were significantly improved from preoperative 46.8 ± 9.2 to 12.2 ± 2.6 at 24 months follow-up in the Rocker group and from preoperative 45.8 ± 9.8 to 11.8 ± 2.4 at 24 months follow-up in the X-Stop group. JOA score also improved significantly in both groups. The radiographic parameters of DHI and FHI in both groups increased immediately postoperatively, however, the improvements seemed to revert toward initial value during follow-up. Two patients in Rocker group demonstrated implant dislocation within one week postoperatively and one patient in X-Stop group demonstrated implant migration at two months postoperatively.

**Conclusions:**

Preliminary clinical and radiographic outcome was similar between Rocker and X-Stop group. For patients of lumbar spinal stenosis with unilateral nerve root involved or mild-to-moderate central canal stenosis, Rocker offers a new alternative with less damage.

## Background

Lumbar spinal stenosis (LSS) is a common disease affected the elderly population. Symptoms exhibit lower back pain, buttock pain, leg pain and neurogenic intermittent claudication [1]. The pathology lies in the compression of the neural elements on the affected level by herniated nucleus pulposus, hyperplastic ligamentum flavum or hypertrophic facet joint leading to central, lateral, or foraminal stenosis [2, 3]. Patients with severe symptoms were recommended to undertake surgery [4, 5].

Recently, interspinous process stabilization (IPS) has been used clinically as a new method for the treatment of LSS. IPS can restore the intervertebral height, increase the foraminal area and the sagittal diameter of lumbar spinal canal and restrict the over-extension of the spine [6, 7]. Two major surgical procedures have been reported for these new devices. IPS could be used alone to distract spinal process to provide an indirect decompression [8, 9]. They could also be implanted combined with microdecompression [10, 11]. Despite concerns about their safety and efficacy, IPS provides a less invasive treatment for patients suffering from LSS and is increasingly used in spine surgery [7, 12].

X-Stop (Medtronic, Minnesota, the United States) is the first IPS that received Food and Drug Administration (FDA) approval in the United States for the treatment of LSS and is most widely used. Since the approval of X-Stop, a number of studies have reported the clinical outcome [13–18]. Rocker (Guoyang, Shanghai, China) is a new type of IPS that is made of polyetherehterketone (PEEK) materials. In contrast to conventional titanium materials, PEEK has near-physiologic elastic modulus and better tissue compatibility [19, 20]. Moreover, owing to its specific self-deploy design, Rocker can be inserted and fixed via unilateral approach. For patients with mild-to-moderate central spinal stenosis or lateral recess stenosis, simple distraction or unilateral microdecompression could be effective. However, most of the IPS available now need to be implanted from both sides of interspinous process and this procedure generates extra damage. So theoretically, IPS via unilateral approach such as Rocker could be more appropriate than traditional ones via bilateral such as X-Stop. To our knowledge, no literature on clinical outcome of Rocker is available now. Therefore, this study aimed to evaluate the clinical safety and efficiency of Rocker compared with X-Stop for lumbar spinal stenosis.

## Methods

### General information

From March 2011 to September 2012, 62 patients with single level LSS were enrolled in this study. Patients enrolled in the study were allocated to the Rocker or the X-Stop group randomly by closed envelopes. The study was approved by the institutional review board of General Hospital of Jinan Military Commanding Region, and the necessary informed consent was obtained from the parents of each patient. The included patients were either mild-to-moderate central canal stenosis that just required distraction without decompression or lateral recess stenosis that required unilateral nerve root decompression. Spondylolisthesis, scoliosis with Cobb angle >10°, vertebrae fractures, severe osteoporosis with T value **<** 2.5, active infection, spinal tumor or ankylosing spondylitis were excluded from this study.

Preoperative evaluation included anterior-posterior, lateral and flexion–extension lumbar spine radiographs, CT and MRI scans. Patients with deformed interspinous process on affected levels detected by X-rays or sagittal CT reconstruction were also excluded.

### Surgical techniques

**Fig. 1:**
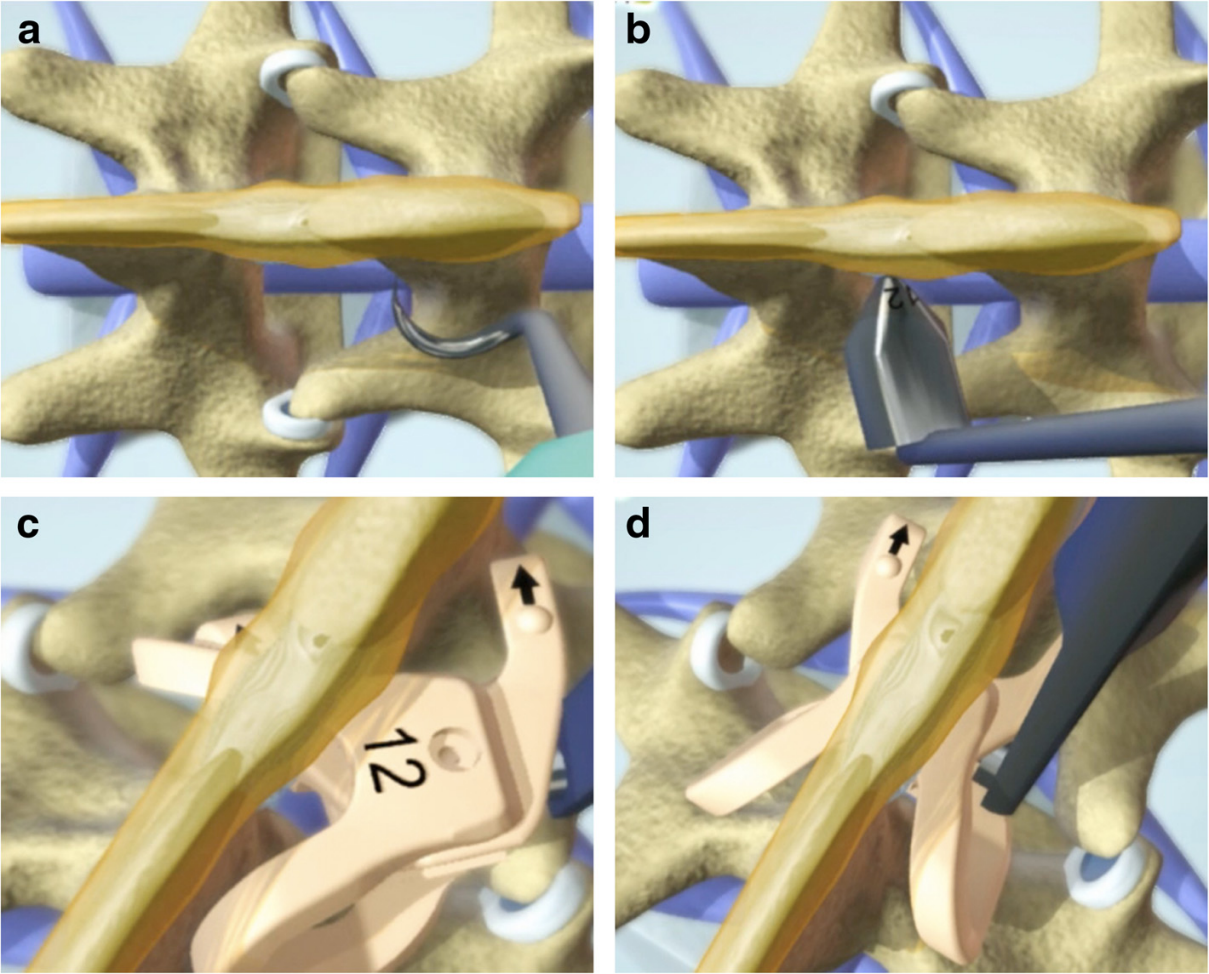
Schematic illustrations demonstrate the surgical procedure to install Rocker. A customized detacher is used to partially dissect the paraspinous muscles of asymptomatic side (**a**). Properly sized pilot is inserted to ensure that the optimal distraction were achieved (**b**). Unlocked Rocker is folded to facilitate installation (**c**). When inserting between spinous processes, folded Rocker deploys and locks by itself (**d**)

All patients were operated by the same team. The patient was positioned on a radiolucent table in the prone position after epidural anesthesia. A posterior midline incision (2–7 cm) was made on the affected level. The supraspinous ligament was exposed and preserved. Then the paraspinous muscles were bluntly dissected from processes and lamina unilaterally for Rocker and bilaterally for X-Stop. For patients with lateral recess stenosis, decompression of the involved nerve root was performed and was completed with discectomy if necessary to ensure adequate relaxation of the nerve root. The small dilator was inserted through interspinous ligament and a customized detacher was used to dissect the paraspinous muscles of asymptomatic side partially to facilitate installation in the Rocker group. Appropriate pilot was placed to ensure that the optimal distraction were achieved. Same size of device was inserted and fixed by screws for X-Stop and by self-lock for Rocker (Fig. 1). Surgical wound was closed in layers and drainage tube was placed for 1–2 days. Patients were allowed to ambulate with waist protections 1–2 days after operation and were requested to wear a waist brace for 12 weeks. Dorsal muscles exercise was recommended one week after operation.

### Clinical outcomes evaluation

The clinical outcome was quantified using Oswestry Disability Index (ODI) score and Japanese orthopaedics association (JOA) score at the following time points: preoperative, postoperative at 1 weeks, 3 months, 6 months, 12 months and 24 months. The minimal clinically important difference (MCID) for ODI was considered to be 8 points [21–23]. The number of patients acquired MCID at the final follow-up was also recorded.

### Radiologic outcomes evaluation

**Fig. 2:**
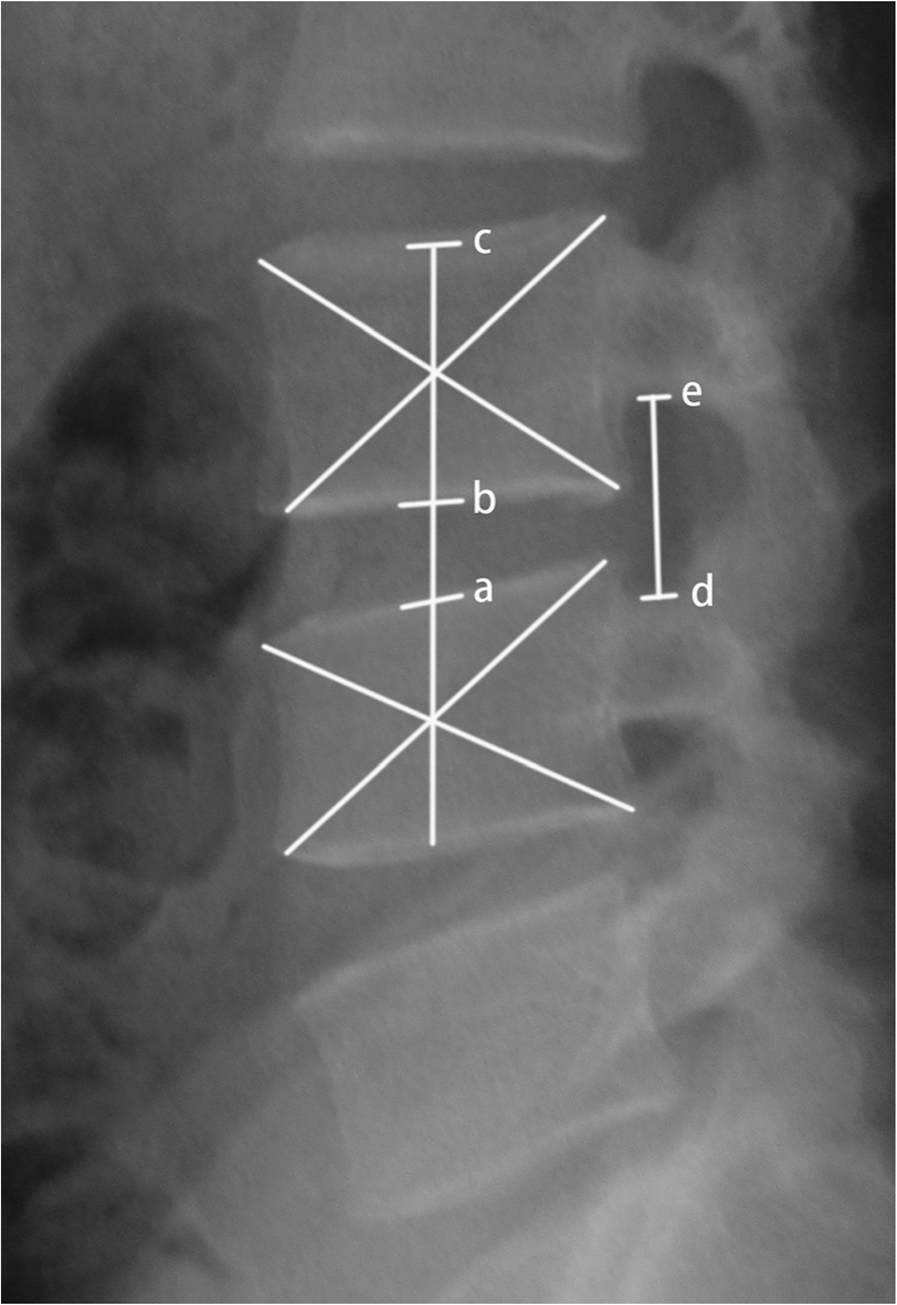
DHI (disc height index) is measured by ab/bc and FHI (foraminal height index) is measured by de/bc. Ab indicates the disc height, bc indicates the vertebrae height and de indicates the longest distance between the upper and lower vertebral pedicle. Ac represents the line labeling the centers. The center of the vertebral body is marked by the crossing point of two diagonal lines

Standard standing radiographs were obtained at each interval. The disc height index (DHI) was measured by Kim’s method (Fig. 2) [24]. Foraminal height index (FHI) was measured by the maximum distance from the lower edge of the upper vertebral pedicle to the upper edge of the lower vertebral pedicle/height of the lower vertebrae (Fig. 2). DHI and FHI were measured by two doctors independently.

### Statistical analysis

SPSS version 13.0 for Windows was used for statistical analyses. The current study was designed to detect the difference of at least 8 points in change on the ODI from baseline to the 24 months follow-up [21–23]. Baseline standard deviation was estimated at 9 points. TypeIerror α was set at 0.05 and typeIIerror β was set at 0.1. Considering these assumptions and adding 10 % for possible drop-outs, we estimated that 60 patients in total were required to complete the study. The independent *t*-test was used to compare parametric data. Categorical variables were compared by *χ*^2^-tests. *p* < 0.05 was considered significantly statistical difference.

## Results

### General information

**Fig. 3:**
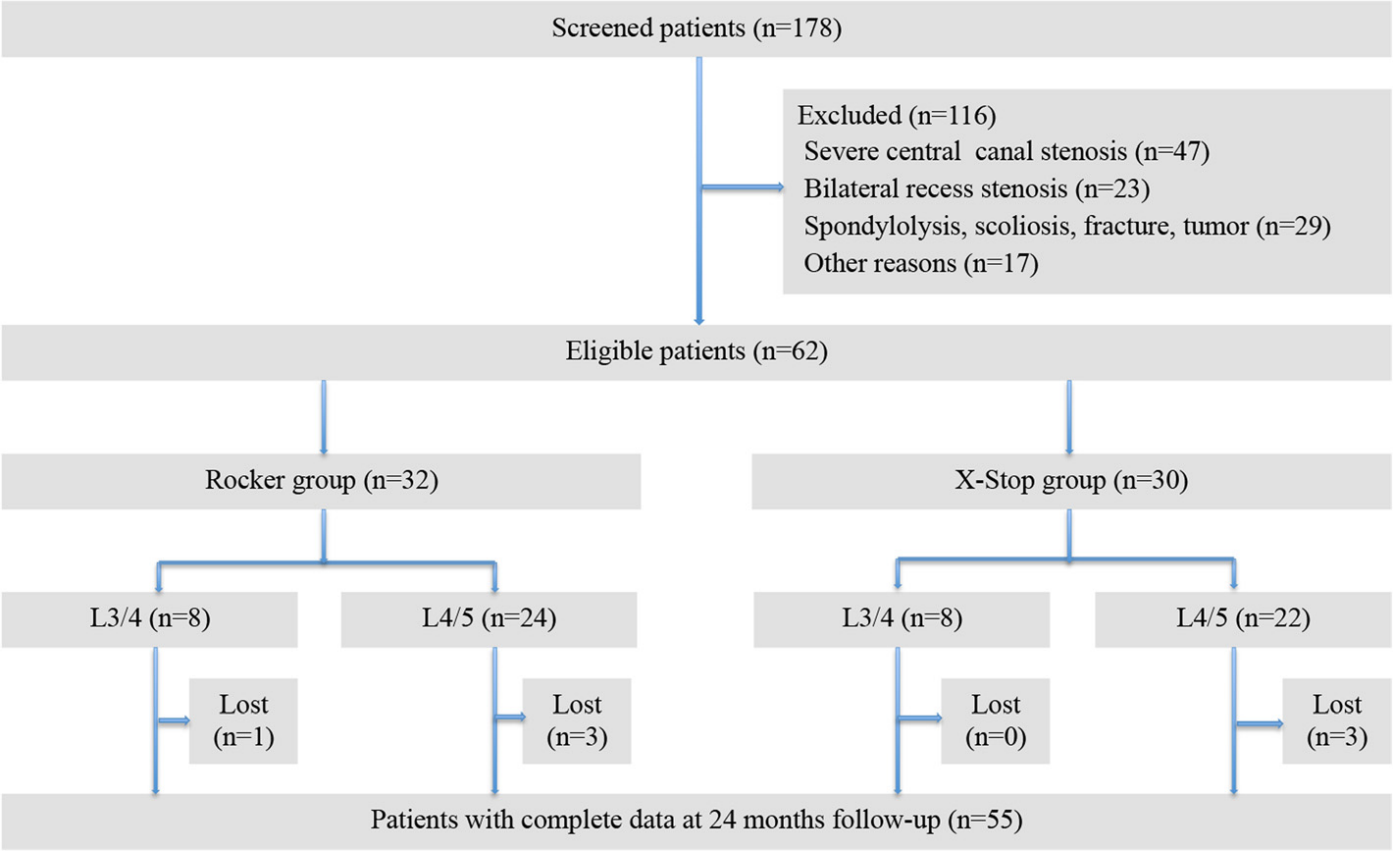
Flow diagram with study enrolment and follow-up

**Fig. 4:**
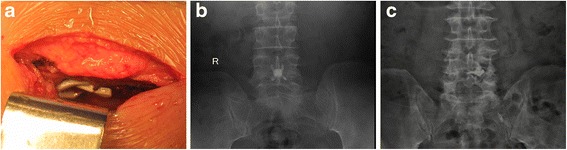
Intraoperative photographs displays that Rocker has been fixed between spinous process (**a**). Anterior to posterior radiographic image shows a properly placed Rocker (**b**). Anterior and posterior radiographic image demonstrates that Rocker dislocated to the paraspinous site (**c**)

Thirty-two patients were treated with Rocker and thirty patients were treated with X-Stop. Figure 3 shows the inclusion and exclusion process. There were one patient died (not surgery related), one patient suffered from spinous process fracture in the Rocker group. Two Rockers migrated within one week in the Rocker group (Fig. 4), while there were two patients lost and one patient with implant dislocation in X-stop group during the follow-up. So 28 patients and 27 patients with complete data during 24-month follow-up were obtained in Rocker and X-Stop group respectively. There were seven patients involved at L3/4 level and 21 patients involved at L4/5 level in the Rocker group, while there were eight patients involved at L3/4 level and 19 patients involved at L4/5 level in the X-Stop group. Baseline characters were presented in Table 1. No significant differences were shown in terms of gender, surgical level, smoking, obesity (body mass index ≥ 30) between groups (Table 1) [25].

**Table 1:** Basic characteristics of the two groups

	Rocker group	X-Stop group	*p* value
Number	28	27	
Mean age (range),years	62.1(52–78)	60.5(50–74)	0.848^a^
Gender(Male/Female)	13/15	15/12	0.498^b^
BMI ≥ 30	6	4	0.729^c^
Current smokers	7	7	0.937^b^
Surgical level			0.700^b^
L3/4	7	8	
L4/5	21	19	
Surgical procedure			0.432^b^
Simple distraction	10	7	
Unilateral decompression	18	20	

*BMI* body mass index

^a^ *p* value from independent samples *t* test

^b^ *p* value from *χ* ^2^-tests

^c^ *p* value from Fisher exact tests

The mean operative time was 53.6 ± 15.0 min (range, 30–90 min) in the Rocker group and 63.1 ± 13.6 min (range, 40–100 min) in the X-Stop group (*p* = 0.421). The average blood loss was 111 ± 71 ml (range, 50 to 400 ml) in the Rocker group and 138 ± 68 ml (range, 50 to 350 ml) in the X-Stop group (*p* = 0.429). The mean duration of hospital stays were 5.5 ± 1.9 and 5.8 ± 1.6 days in the Rocker and the X-Stop group, respectively. There was one patient with intraoperative dural rupture in the Rocker group. Two Rockers unlocked and displaced within one week postoperatively detected by X-ray. Revision surgery was performed to implant a new Rocker in one patient and install pedicle screw systems in the other patient as soon as the diagnosis was made. One patient demonstrated X-Stop dislocation with low back pain and abnormal sound during activities at 2-month postoperatively. Implant was removed and pedicle screws fixation was performed for the patient.

### Clinical outcome

**Fig. 5:**
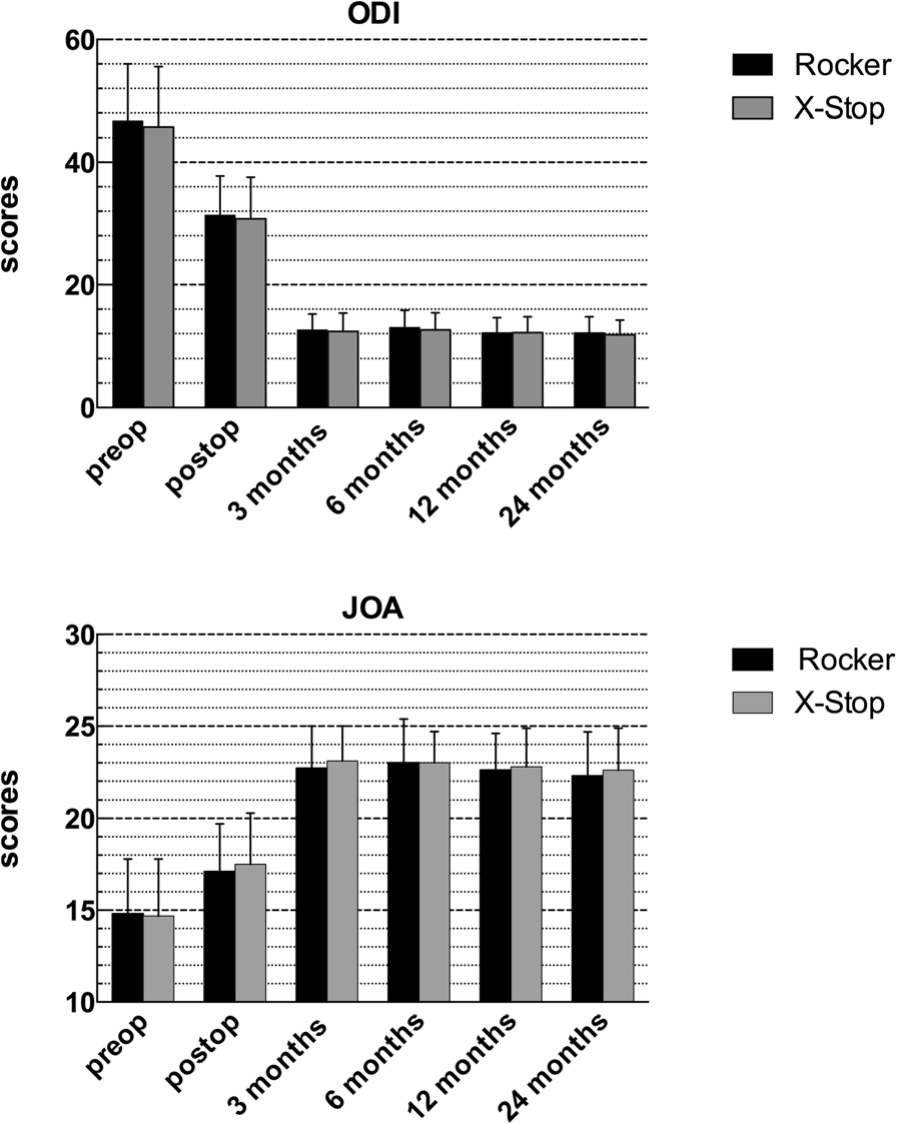
Clinical outcome measured by ODI (Oswestry disability index) score and JOA (Japanese orthopaedics association) score in both groups

Clinical ODI and JOA score were significantly improved at 1 week, 3 months, 6 months, 12 months and 24 months postoperatively as compared with preoperative score in both groups (*p* < 0.05), with similar outcome observed at each interval postoperatively between the two groups (*p >* 0.05) (Fig. 5). The mean ODI score was significantly improved from preoperative 46.8 ± 9.2 to 12.2 ± 2.6 at 24 months follow-up in the Rocker group and from preoperative 45.8 ± 9.8 to 11.8 ± 2.4 at 24 months follow-up in the X-Stop group. Among the 55 patients with complete data during follow-up, 49 achieved a MCID.

### Radiographic outcome

**Fig. 6:**
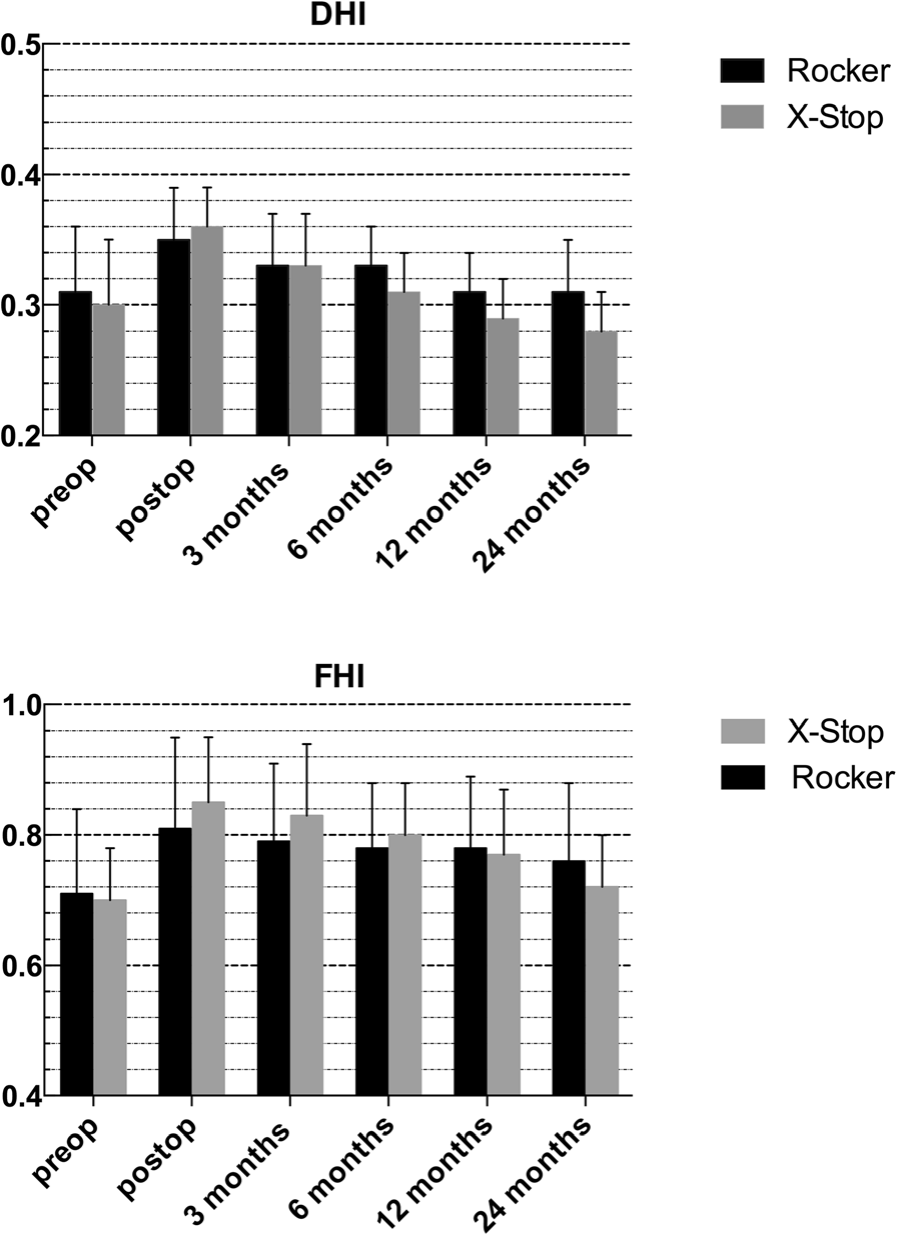
Radiographic outcome of DHI (disc height index) and FHI (foraminal height index) in both groups

Preoperative DHI and FHI had no statistical difference. DHI and FHI in both groups increased after operation without statistical significance. However, they showed a gradually decline within 2 years (Fig. 6). The mean lordosis at the index level were 5.2 ± 2.9° preoperatively and 3.1 ± 2.2°at final follow-up in the Rocker group, while 5.5 ± 3.3°preoperatively and 3.6 ± 2.4°at final follow-up in the X-Stop group.

## Discussion

The current research studied clinical and radiographic outcome of a novel IPS Rocker for the LSS via unilateral approach compared with a widely used IPS X-Stop. The two IPS had favorable clinical outcome.

Laminectomy was an effective and to some extent a standard treatment for LSS reported by Weinstein et al. [5]. In recent years, microdecompression has a growing tendency. A recently released study by Nerland et al. showed that microdecompression had equivalent outcomes to laminectomy for LSS [21]. This study supported the effectiveness of microdecompression powerfully. As the primary clinical outcome, ODI score in the current study was compared with the two famous studies. In contrast to the two studies, the mean ODI score in the current study was higher preoperatively and lower postoperatively. However, the difference did not mean that interspinous process stabilization in the current study could achieve better clinical outcomes. Firstly, the current study was conducted in China, which is a developing country with a large population. Health care system in China is impoving year by year but still not as well developed as that in the Unite States and Norway. Many patients will not seek medical advice until their symptoms turn serious. This could be the reason why preoperative ODI score was higher in the current study. Secondly, regarding to postoperative lower ODI score, due to different cultural background, people in China are relatively conservative and most of them feel reluctant to answer sex life questions, which might lead to ODI-8 answered inappropriately and generate bias. Besides, as Saberi et al. reported, we speculated that higher preoperative ODI might be associated with better surgical outcome [26].

It should be noted that different surgical procedures have been reported for IPS. They could be used alone without decompression procedure and just provided an indirect decompression by spinal process distraction [8, 9]. There were also studies that reported the combination of microdecompression and IPS [10, 11]. In the current study, the majority of the enrolled patients were combined with microdecompression. The clinical results in this study were consistent with those in the study by Ploumis et al. [11] and the combination was believed to provide indirect decompression for the nerve root on the nonoperative side,unload the disc and stabilize the spine [10].

Reoperation rate after IPS implantation is another widely concerned issue. Three patients were reoperated in the current study. Higher risk of secondary operation by X-Stop was reported by LØnne [27] and Stromqvist [8]. However, when comparing the two studies with the current studies, we found that the dominant reason for reoperation in the two studies was persistent or recurrent symptoms. Among the 13 patients reoperated in the study by Stromqvist, 11 patients were due to persistent symptoms after the first operation and two patients were due to recurrent symptoms [8], while among the 13 patients reoperated in the study by LØnne, four patients were due to persistent symptoms after the first procedure, six patients were due to recurrent symptoms, the remaining three patients were due to implant dislocation or fracture of spinal process [9]. Obviously, ineffectiveness was the dominant cause for secondary operation in Stromqvist and LØnne’s studies. However, it must be noted that all the patients enrolled in the X-Stop groups in the two studies underwent indirect decompression by spinal process distraction using X-Stop alone without direct decompression for nerve root or central canal. Whereas in the current study, the majority of the patients underwent IPS combined with microdecompression. No reoperation due to persistent or recurrent symptoms occurred during follow-up in the current study. We speculated that the combination of microdecompression for majority of patients in this study might account for the different reoperation rates between the previous two studies and the current study. However, it needs for further investigation.

IPS are advocated to restore the disc height and foraminal height. In Ryu’s comparative study, 16 patients with DIAM showed that mean DHI was 11.3 before operation, 12.7 on the first postoperative day and 11.2 at one year follow-up and FHI was 19.2 before operation, 20.1 at 6-month follow-up and 20.0 at one year follow-up [28]. Zhou et al. [29] reported 23 patients with lumbar degenerative diseases treated with in-space system. Mean foraminal height was preoperative 18.7 mm, 21.4 mm at two weeks postoperatively and 21.1 mm at 18 months postoperatively.

In the current study, DHI had a 13 % increase immediately postoperatively and gradually declined to the preoperative level at 24-month follow-up in the Rocker group. In the X-Stop group, DHI had a 20 % increase immediately postoperatively and gradually declined below the preoperative level. FHI had a similar trend like DHI. Despite no statistical significance, it seems that PEEK material Rocker maintains improvement a little longer. Some previous studies have also reported that PEEK IPS displayed favorable radiographic changes. Fifty patients with lumbar degenerative disease treated by Wallis showed that intervertebral disc height and the neural foramina height at 12 and 24 months after surgery were significantly higher than that before surgery [30]. Sandu et al. [31] observed disc rehydration in a case series after Wallis implantation on postoperative MRI scans. However, some other studies presented the opposite view. A recent experimental animal study conducted by Barz et al. [32] detected obvious resorption of the spinous processes at the site of the PEEK IPS. This histomorphometric changes in their BB.4S rat models indicated that PEEK interspinous devices like the Wallis might have time-limited effects. Likewise, a retrospective study by Sobottke et al. [33] demonstrated the softer IPS Wallis and Diam displayed more significantly postoperative radiological changes toward the initial values than X-Stop. Sobottke speculated that softer implant breakdown led to the difference. So up to now, previous studies have not reached a consensus. From our perspective, it is not possible to completely transfer results from the rat model to the adult human in Barz’s research and comparative radiographic outcome had no statistical significance in Sobottke’s series. Further studies with larger sample and longer follow-up or basic research are required to settle this issue.

By virtue of its dedicate self-deploy design, Rocker can be inserted and locked via unilateral approach. The installation procedure is simple and quick. In this series, less blood loss and shorter operation time were observed in the Rocker group. What’s more, unilateral approach reduced unnecessary damage. Traction of paraspinal muscle and excessive dissection can lead to denervation, atrophy and irreversible muscle injury and ultimately result in persistent low back pain [34, 35].

It should be taken seriously that two Rockers unlocked and displaced within one week postoperatively. There may be two reasons for these complications. Firstly, when installing, forces should be applied in parallel to the rotation axis of Rocker in order that Rocker is deployed and locked correctly. Because of the existed tension between spinous process, Rocker may be fixed apparently rather than locked actually. In this case, dislocation is definitely inevitable. So it must be confirmed that Rocker is properly locked before the wound is closed. A clicking sound that indicates successful lock-up can be helpful, but it should be paid attention to that the special sound is not always clear due to the tension between spinous process. In our experience, the most effective way to confirm a stable lockup is that the Rocker can not be fold by pushing the upper and lower ends. Secondly, we find that under violence in vitro, the self-lock system does not seems to be sufficiently sturdy and can be unlocked. In the early stage after operation, dissection and traction of paraspinal muscle during operation lead to a relatively poor stability in lumbar spine and less support on the sides of Rocker. This may also partly result in unlock and dislocation in the early stage. So a definite lock-up and less activity in the early stage may be helpful to prevent implant failure.

The current study demonstrated the clinical feasibility and validity of Rocker IPS. Compared with X-Stop, Rocker has similar clinical effects and possibly superior restoration of DHI and FHI. Moreover, Rocker is less invasive in contrast to X-Stop. However, surgeons should install Rocker correctly to ensure a successful lock-up and modified design in locking system aimed to resist larger load may be beneficial to prevent early dislocation.

### Study strengths and limitations

The results in the present study were strengthened by prospective data collection, controlled group and specific inclusion–exclusion criteria. There were some limitations that should also be considered. First, although the reliability and validity of the JOA scale have been testified [36], it is mostly used in Asian countries and is not a measurement as conventional as ODI across the world. Second, the loss to follow-up was 11.3 % (7/62) at the final follow-up, which was relatively high.

## Conclusions

This study demonstrated that Rocker and X-Stop group have similar preliminary clinical and radiographic outcome. For patients of lumbar spinal stenosis with unilateral nerve root involved or mild-to-moderate central canal stenosis, Rocker offers a new alternative with less damage.

## Abbreviations

IPS: Interspinous process stabilization
ODI: Oswestry disability index
JOA: Japanese orthopaedics association
DHI: Disc height index
FHI: Foraminal height index
MCID: Minimal clinical important difference
LSS: Lumbar spinal stenosis
FDA: Food and drug administration
PEEK: Bolyetherehterketone
VAS: Visual analogue scale
RDQ: Roland disability questionnaire

## References

[1] Ishimoto Y, Yoshimura N, Muraki S, Yamada H, Nagata K, Hashizume H (2013). Associations between radiographic lumbar spinal stenosis and clinical symptoms in the general population: the Wakayama Spine Study. Osteoarthr Cartilage.

[2] Hasegawa T, An HS, Haughton VM, Nowicki BH (1995). Lumbar foraminal stenosis: critical heights of the intervertebral discs and foramina. A cryomicrotome study in cadavera. J Bone Joint Surg Am.

[3] Yoshida M, Shima K, Taniguchi Y, Tamaki T, Tanaka T (1992). Hypertrophied ligamentum flavum in lumbar spinal canal stenosis. Pathogenesis and morphologic and immunohistochemical observation. Spine (Phila Pa 1976).

[4] Kovacs FM, Urrutia G, Alarcon JD (2011). Surgery versus conservative treatment for symptomatic lumbar spinal stenosis: a systematic review of randomized controlled trials. Spine (Phila Pa 1976).

[5] Weinstein JN, Tosteson TD, Lurie JD, Tosteson AN, Blood E, Hanscom B (2008). Surgical versus nonsurgical therapy for lumbar spinal stenosis. N Engl J Med.

[6] Grasso G, Giambartino F, Iacopino DG (2014). Clinical analysis following lumbar interspinous devices implant: where we are and where we go. Spinal Cord.

[7] Gazzeri R, Galarza M, Alfieri A (2014). Controversies about interspinous process devices in the treatment of degenerative lumbar spine diseases: past, present, and future. Biomed Res Int.

[8] Stromqvist BH, Berg S, Gerdhem P, Johnsson R, Moller A, Sahlstrand T (2013). X-Stop versus decompressive surgery for lumbar neurogenic intermittent claudication: randomized controlled trial with 2-year follow-up. Spine (Phila Pa 1976).

[9] Lonne G, Johnsen LG, Rossvoll I, Andresen H, Storheim K, Zwart JA (2015). Minimally invasive decompression versus X-Stop in lumbar spinal stenosis: a randomized controlled multicenter study. Spine (Phila Pa 1976).

[10] Fuchs PD, Lindsey DP, Hsu KY, Zucherman JF, Yerby SA (2005). The use of an interspinous implant in conjunction with a graded facetectomy procedure. Spine (Phila Pa 1976).

[11] Ploumis A, Christodoulou P, Kapoutsis D, Gelalis I, Vraggalas V, Beris A (2012). Surgical treatment of lumbar spinal stenosis with microdecompression and interspinous distraction device insertion. A case series. J Orthop Surg Res.

[12] Puzzilli F, Gazzeri R, Galarza M, Neroni M, Panagiotopoulos K, Bolognini A (2014). Interspinous spacer decompression (X-STOP) for lumbar spinal stenosis and degenerative disk disease: a multicenter study with a minimum 3-year follow-up. Clin Neurol and Neurosur.

[13] Kuchta J, Sobottke R, Eysel P, Simons P (2009). Two-year results of interspinous spacer (X-Stop) implantation in 175 patients with neurologic intermittent claudication due to lumbar spinal stenosis. Eur Spine J.

[14] Siddiqui M, Smith FW, Wardlaw D (2007). One-year results of X STOP interspinous implant for the treatment of lumbar spinal stenosis. Spine.

[15] Nandakumar A, Clark NA, Smith FW, Wardlaw D (2013). Two-year results of X-Stop interspinous implant for the treatment of lumbar spinal stenosis a prospective study. J Spinal Disord Tech.

[16] Hartjen CA, Resnick DK, Hsu KY, Zucherman JF, Hsu EH, Skidmore GA. Two-Year Evaluation of the X-STOP Interspinous Spacer in Different Primary Patient Populations With Neurogenic Intermittent Claudication due to Lumbar Spinal Stenosis. J Spinal Disord Tech*.* 2013. [Epub ahead of print] 10.1097/BSD.0b013e31827b671f 23168396

[17] Burnett MG, Stein SC, Bartels R (2010). Cost-effectiveness of current treatment strategies for lumbar spinal stenosis: nonsurgical care, laminectomy, and X-STOP Clinical article. J Neurosurg Spine.

[18] Patil S, Burton M, Storey C, Glenn C, Marino A, Nanda A (2013). Evaluation of interspinous process distraction device (X-STOP) in a representative patient cohort. World Neurosurg.

[19] Kurtz SM, Devine JN (2007). PEEK biomaterials in trauma, orthopedic, and spinal implants. Biomaterials.

[20] Toth JM, Wang M, Estes BT, Scifert JL, Seim HB, Turner AS (2006). Polyetheretherketone as a biomaterial for spinal applications. Biomaterials.

[21] Nerland US, Jakola AS, Solheim O, Weber C, Rao V, Lonne G (2015). Minimally invasive decompression versus open laminectomy for central stenosis of the lumbar spine: pragmatic comparative effectiveness study. BMJ.

[22] Brox JI, Reikeras O, Nygaard O, Sorensen R, Indahl A, Holm I (2006). Lumbar instrumented fusion compared with cognitive intervention and exercises in patients with chronic back pain after previous surgery for disc herniation: a prospective randomized controlled study. Pain.

[23] Hellum C, Johnsen LG, Storheim K, Nygaard OP, Brox JI, Rossvoll I (2011). Surgery with disc prosthesis versus rehabilitation in patients with low back pain and degenerative disc: two year follow-up of randomised study. BMJ.

[24] Kim KT, Park SW, Kim YB (2009). Disc height and segmental motion as risk factors for recurrent lumbar disc herniation. Spine (Phila Pa 1976).

[25] Flegal KM, Carroll MD, Kit BK, Ogden CL (2012). Prevalence of obesity and trends in the distribution of body mass index among US adults, 1999–2010. JAMA.

[26] Saberi H, Isfahani AV (2008). Higher preoperative Oswestry disability Index is associated with better surgical outcome in upper lumbar disc herniations. Eur Spine J.

[27] Lonne G, Johnsen LG, Aas E, Lydersen S, Andresen H, Ronning R (2015). Comparing cost-effectiveness of X-Stop with minimally invasive decompression in lumbar spinal stenosis: a randomized controlled trial. Spine (Phila Pa 1976).

[28] Ryu SJ, Kim IS (2010). Interspinous implant with unilateral laminotomy for bilateral decompression of degenerative lumbar spinal stenosis in elderly patients. J Korean Neurosurg S.

[29] Zhou D, Nong LM, Du R, Gao GM, Jiang YQ, Xu NW (2013). Effects of interspinous spacers on lumbar degenerative disease. Exp Ther Med.

[30] Pan B, Zhang ZJ, Lu YS, Xu WG, Fu CD (2014). Experience with the second-generation Wallis interspinous dynamic stabilization device implanted in degenerative lumbar disease: a case series of 50 patients. Turk Neurosurg.

[31] Sandu N, Schaller B, Arasho B, Orabi M (2011). Wallis interspinous implantation to treat degenerative spinal disease: description of the method and case series. Expert Rev Neurother.

[32] Barz T, Lange J, Melloh M, Staub LP, Merk HR, Kloting I (2013). Histomorphometric and radiographical changes after lumbar implantation of the PEEK nonfusion interspinous device in the BB.4S rat model. Spine.

[33] Sobottke R, Schluter-Brust K, Kaulhausen T, Rollinghoff M, Joswig B, Stutzer H (2009). Interspinous implants (X Stop (R), Wallis (R), Diam (R)) for the treatment of LSS: is there a correlation between radiological parameters and clinical outcome?Eur. Spine J.

[34] Sihvonen T, Herno A, Paljarvi L, Airaksinen O, Partanen J, Tapaninaho A (1993). Local denervation atrophy of paraspinal muscles in postoperative failed back syndrome. Spine (Phila Pa 1976).

[35] Gejo R, Matsui H, Kawaguchi Y, Ishihara H, Tsuji H (1999). Serial changes in trunk muscle performance after posterior lumbar surgery. Spine (Phila Pa 1976).

[36] Fujiwara A, Kobayashi N, Saiki K, Kitagawa T, Tamai K, Saotome K (2003). Association of the Japanese Orthopaedic Association score with the Oswestry Disability Index, Roland-Morris Disability Questionnaire, and short-form 36. Spine (Phila Pa 1976).

